# Transitions into and out of daylight saving time compromise sleep and the rest-activity cycles

**DOI:** 10.1186/1472-6793-8-3

**Published:** 2008-02-12

**Authors:** Tuuli A Lahti, Sami Leppämäki, Jouko Lönnqvist, Timo Partonen

**Affiliations:** 1 Department of Mental Health and Alcohol Research, National Public Health Institute, Helsinki, Finland; 2 Department of Psychiatry, Helsinki University Central Hospital, Helsinki, Finland; 3 Department of Psychiatry, University of Helsinki, Helsinki, Finland

## Abstract

**Background:**

The aim of this study was to analyze the effects of transition out of and into daylight saving time on the rest-activity cycles and sleep. Rest-activity cycles of nine healthy participants aged 20 to 40 years were measured around transitions out of and into daylight saving time on fall 2005 and spring 2006 respectively. Rest-activity cycles were measured using wrist-worn accelerometers. The participants filled in the Morningness-Eveningness and Seasonal Pattern Assessment Questionnaires before starting the study and kept a sleep diary during the study.

**Results:**

Fall transition was more disturbing for the more morning type and spring transition for the more evening type of persons. Individuals having a higher global seasonality score suffered more from the transitions.

**Conclusion:**

Transitions out of and into daylight saving time enhanced night-time restlessness and thereby compromised the quality of sleep.

## Background

Daylight saving time (DST) is commonly used worldwide and affects millions of people annually. It equals to one-hour time zone crossing eastward in the spring and westward in the fall. In the European Union, DST currently begins on the last Sunday of March, when the clocks are turned forwards by one hour, and ends on the last Sunday of October, when the clocks are turned backwards by one hour. The rationale for DST is to improve the match between the daylight hours with the activity peaks of a population. Fall transition out of DST increases the available daylight in the morning by one hour. Spring transition into DST leads to an increase of the available daylight in the evening. In our previous studies, we found that transition into daylight saving time may disrupt the rest-activity cycle in healthy adults [1,2]. Herein, our aim was to assess the daily rest-activity cycles together with night-time sleep at transitions out of and into daylight saving time in healthy adults. Our goal was to find out whether the changes induced by transition into DST were similar in fall and spring.

## Results

### Fall: before versus after transition

The movement and fragmentation index (P = 0.01; Z = -2,52) was increased in all the participants after the transition (Table 1). Sleep efficiency (P = 0.02; Z = -2.38) and relative amplitude (P = 0.02; Z = -2.43) were reduced in all except one participant after transition.

**Table 1:** Actigraphic data on sleep and the rest-activity cycles from fall 2005 (before versus after DST transition).

FALL	Mean/before	95% confidence interval/before	Standard deviation/before	Mean/after	95% confidence interval/after	Standard deviation/after	Significance
Sleep efficiency (%)	86.33	80.80 – 91.87	6.62	79.76	74.79 – 84.72	5.94	0.006
Movement and fragmentation index	23.13	16.50 – 29.75	7.92	35.66	26.02 – 43.30	9.14	0.003
Relative amplitude	0.93	0.90 – 0.96	0.36	0.85	0.74 – 0.96	0.14	0.055
Intra-daily stability	0.58	0.47 – 0.70	0.15	0.57	0.42 – 0.72	0.19	0.880
Intra-daily variability	0.83	0.73 – 0.93	0.13	0.82	0.67 – 0.97	0.19	0.857

### Spring: before versus after transition

The movement and fragmentation index (P = 0.01; Z = -2.52) was increased after transition (Table 2). Sleep efficiency was not reduced significantly after the spring transition.

**Table 2:** Actigraphic data on sleep and the rest-activity cycles from spring 2006 (before versus after DST transition).

SPRING	Mean/before	95% confidence interval/before	Standard deviation/before	Mean/after	95% confidence interval/after	Standard deviation/after	
Sleep efficiency (%)	87.29	83.26 – 91.23	4.82	83.78	76.51 – 91.06	8.70	0.161
Movement and fragmentation index	20.93	15.82 – 26.05	6.12	28.68	18.82 – 38.55	11.80	0.019
Relative amplitude	0.94	0.92 – 0.96	0.03	0.91	0.88 – 0.95	0.05	0.140
Intra-daily stability	0.64	0.53 – 0.76	0.15	0.62	0.49 – 0.75	0.17	0.313
Intra-daily variability	0.92	0.76 – 1.10	0.20	0.84	0.66 – 1.02	0.24	0.354

### Fall before versus spring before

To see whether there was any difference in the baseline conditions, we compared the scores before the two transitions. There was none.

### Sleep and rest-activity cycles in relation to MES and GSS

**Figure 1:**
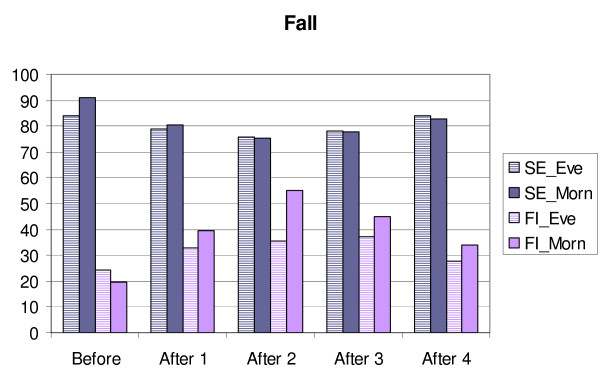
**Changes in sleep efficiency (SE) and movement and fragmentation index (FI) in MES-subgroups/Fall.** Before = mean for four days before transition, After 1,2,3 and 4 = means for the days after transition. Eve = MES-subgroup evening type, Morn = MES-subgroup morning type.

**Figure 2:**
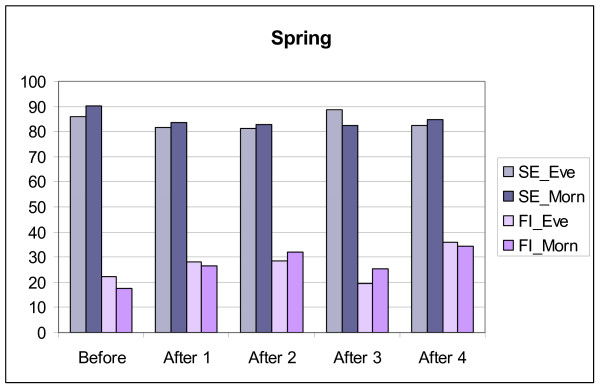
**Changes in sleep efficiency (SE) and movement and fragmentation index (FI) in MES-subgroups/Spring.** Before = mean for four days before transition, After 1,2,3 and 4 = means for the days after transition. Eve = MES-subgroup evening type, Morn = MES-subgroup morning type.

**Figure 3:**
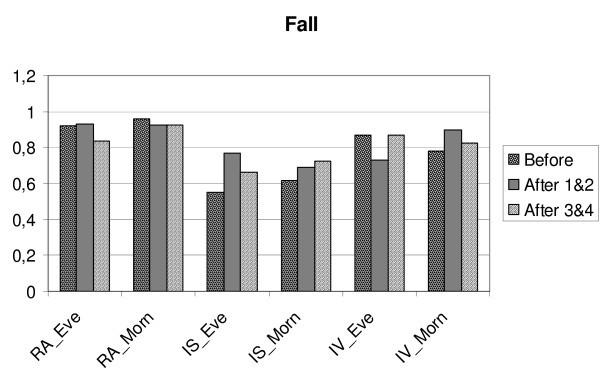
**Changes in relative amplitude (RA), intra-daily stability (IS) and intra-daily variability (IV) in MES-subgroups/Fall. ** Before = mean for four days before transition, After 1&2 is mean for two days after transition, After 3&4 = is mean for the days three and four after transition. Eve = MES-subgroup evening type, Morn = MES-subgroup morning type.

**Figure 4:**
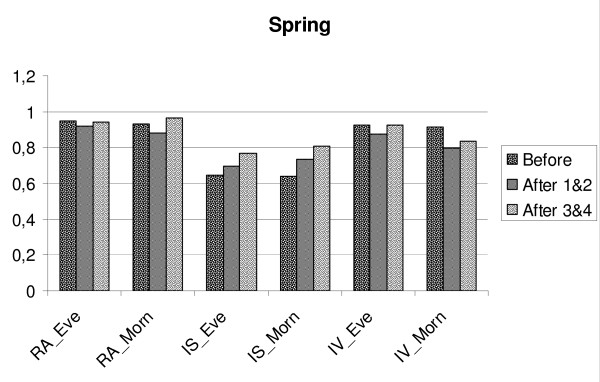
**Changes in relative amplitude (RA), intra-daily stability (IS) and intra-daily variability (IV) in MES-subgroups/Fall.** Before = mean for four days before transition, After 1&2 is mean for two days after transition, After 3&4 = is mean for the days three and four after transition. Eve = MES-subgroup evening type, Morn = MES-subgroup morning type.

**Figure 5:**
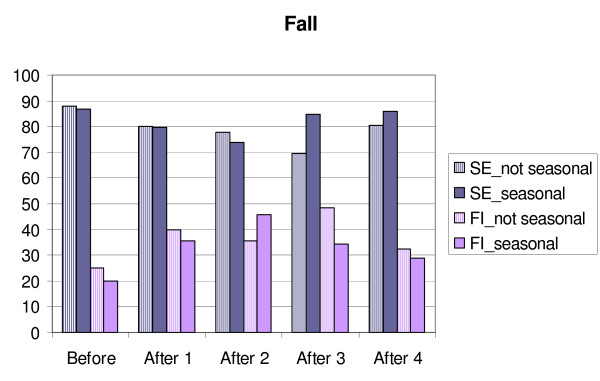
**Changes in sleep efficiency (SE) and movement and fragmentation index (FI) in GSS-subgroups/Fall. ** Before = mean for four days before transition, After 1,2,3 and 4 = means for the days after transition. Not seasonal = GSS-subgroup not seasonal, seasonal = GSS-subgroup seasonal.

**Figure 6:**
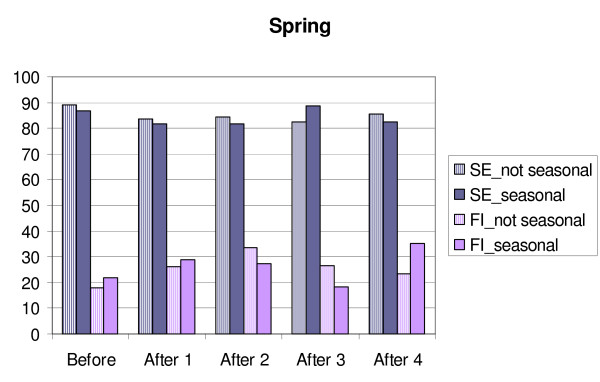
**Changes in sleep efficiency (SE) and movement and fragmentation index (FI) in GSS-subgroups/Fall.** Before = mean for four days before transition, After 1,2,3 and 4 = means for the days after transition. Not seasonal = GSS-subgroup not seasonal, seasonal = GSS-subgroup seasonal.

**Figure 7:**
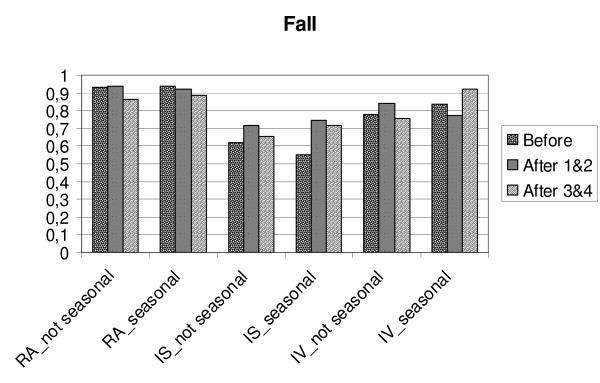
**Changes in relative amplitude (RA), intra-daily stability (IS) and intra-daily variability (IV) in GSS-subgroups/Fall. **Before = mean for four days before transition, After 1&2 is mean for two days after transition, After 3&4 = is mean for the days three and four after transition. Not seasonal = GSS-subgroup not seasonal, seasonal = GSS-subgroup seasonal.

**Figure 8:**
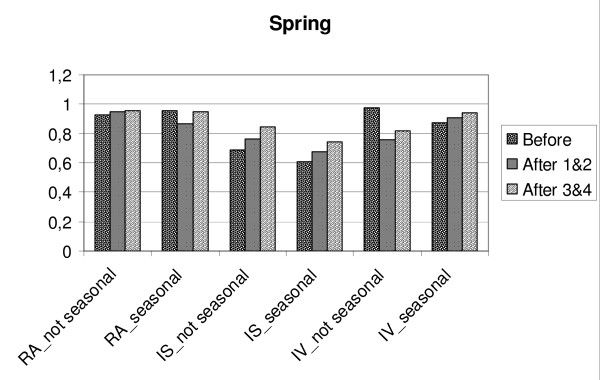
**Changes in relative amplitude (RA), intra-daily stability (IS) and intra-daily variability (IV) in GSS-subgroups/Spring.** Before = mean for four days before transition, After 1&2 is mean for two days after transition, After 3&4 = is mean for the days three and four after transition. Not seasonal = GSS-subgroup not seasonal, seasonal = GSS-subgroup seasonal.

For the participants, the mean (SD and 95% CI) MES was 53.00 (6.75 and 47.82 to 58.18) and the mean (SD and 95% CI) GSS was 9.11 (5.75 and 4.69 to 13.53). Adjustments to transitions out of and into daylight saving time were not accurate or complete four days afterwards (see Figures 1, 2, 3, 4, 5, 6, 7 and 8). The spring transition was more harmful for the more evening type of persons, as the intra-daily stability was reduced more (P = 0.02; r = -0.80) among those with a lower MES. On fall such effect was not seen. Both transitions were more harmful for those persons having greater seasonal changes in mood and behavior. After the fall transition, the movement and fragmentation index was increased more (P < 0.05; r = -0.72) among those with a higher GSS.

## Discussion

Our main finding was that transitions into and out of daylight saving time disrupted night-time sleep. The movement and fragmentation index was increased significantly after both transitions, on average by 54% in fall and 37% in spring. There is a small reservation while using movement and fragmentation index as it still is slightly unclear parameter [3]. However our experience is that movement and fragmentation index reflects well the quality of sleep. Unexpectedly, sleep efficiency was reduced after the transition out of DST only. The durations of slow-wave sleep stages tends to decrease and those of rapid-eye-movement sleep to increase during winter [4]. Transitions out of DST may affect sleep efficiency more than those into DST due to these underlying changes in sleep stages.

Moreover, the relative amplitude of the daily rest-activity cycles was decreased after the transition out of DST, not after the transition into DST. This may bear relevance to the circadian pacemaker whose function is affected by physical exercise and sleep stages [5,6]. Shortage of daylight in the morning during winter challenges the regulation of the circadian clockwork and keeps favoring its natural propensity for phase delays. This shortfall can be overcome by the increased responsiveness to light exposure [7] but may not be counteracted effectively enough among individuals predisposed to depressive disorder [8]. We found herein that individuals having higher global seasonality scores had more disruptions in their rest-activity cycles after the transitions. They may thus be more predisposed to such changes in general but in particular under conditions which challenge the circadian pacemaker, e.g. after shift work schedules or time zone crossings.

We found that the transition out of DST was more detrimental to individuals with the preference to morning activities. Also Kantermann et al. noticed in their recent study that the timing of activity does not adjust to the DST imposition in spring, especially in late chronotypes [9]. This was unexpected, since the fall transition brings one more hour light to the mornings. However, this option to have earlier light exposure may not materialize at all or not be enough to maintain the daily rest-activity cycle and night-time sleep undisturbed. To visualize, the sunrise to sunset times were 8:35 to 17:32 prior to the transition, and 7:38 to 16:29 one day and 7:40 to 16:26 two days after the transition.

In contrast, the transition into DST affected more those with the preference to evening activities. This may have been due to the longer exposure to light in the evening which is known to delay the phase position of the circadian rhythms and their subsidiary rest-activity cycles. To visualize, the sunrise to sunset times were 6:07 to 18:46 prior the transition, and 7:04 to 19:49 one day and 7:01 to 19:51 two days after the transition.

Our findings herein on healthy individuals now show that night-time sleep and the rest-activity cycles are compromised after DST transitions. Earlier, it has been demonstrated that even moderate changes in the timing of the sleep-wake cycle may have profound effects on subsequent mood in healthy young persons [10]. The impact of these twice-a-year transitions on night-time sleep and the daily rest-activity cycles may be more detrimental among patients with mood disorders or circadian rhythm sleep disorders. Therefore, our findings need replication and extension on bigger as well as clinical samples.

Both animal and human studies have demonstrated that the principal clock can be reset with light exposure. Light exposure in the morning (as after fall transitions) advances the phase position of circadian rhythms, whereas light exposure in the evening (as after spring transitions) delays the phase position [11]. The principal clock generating the endogenous rhythms is located in the suprachiasmatic nuclei of the anterior hypothalamus in the brain [12,13]. Light-dark transitions are the most important time-giver for this clock. Optimal sleep quality is achieved when the desired sleep time is aligned with the timing of the endogenous circadian rhythm of sleep and wake propensity [14].

## Conclusion

Transitions out of and into daylight saving time enhance night-time restlessness and compromise the quality of sleep. They may thereby affect mood in a negative way and be a concern for individuals with mood disorder in particular.

## Methods

For the study we had nine actigraphs. The study participants, aged 20–40, were eight women and one man. They were healthy and free of psychotropic medication. All were living in the capital area of Finland (60°12'N), and none was shift-worker nor crossed time zones during the study. All the participants gave a written informed consent. Participants were asked to retain their normal and regular daily schedule during the study. Identical measurement protocols were carried out twice on the same individuals, each wearing an exclusive accelerometer or actigraph (Actiwatch-Plus^®^, Cambridge Neurotechnology Ltd, Cambridgeshire, UK) throughout both study periods.

In fall 2005, DST was started 30 October at 3 a.m. Rest-activity cycles were measured for a period from 24 October to 3 or 10 November, thus yielding data for one week before and one (6 participants) or two (3 participants) weeks after the transition. In spring 2006, DST was started on 26 March at 3 a.m. Rest-activity cycles were measured for a period from 20 March to 3 April, thus yielding data for one week before and one week after the transition. The participants wore the units for all the time, except during short non-waterproof activities. The units were mounted in the non-dominant wrist and positioned using a standardized protocol, recording the intensity, amount and duration of movement in all directions over 0.05 g, with the sampling epoch of 30 sec. The sampling frequency of the units was 32 Hz at maximum, the filters being set from 3 to 11 Hz.

The participants filled in the Morningness-Eveningness Questionnaire [15]. Morningness-Evenigness Questionnaire (MEQ) is a self-report instrument for the assessment of the preference for the daily activity patterns whose sum yields the Morningness-Eveningness Score (MES), ranging from 16 to 86. The highest score indicates the definite preference of activities in the morning (morningness), whereas the lowest one indicates the definite preference of activities in the evening (eveningness). Participants also filled in the Seasonal Pattern Assessment Questionnaire [16]. Seasonal Pattern Assessment Questionnaire (SPAQ) is a self-report instrument for the assessment of the seasonal changes in the length of sleep, social activity, mood, weight, appetite, and energy whose sum yields the Global Seasonality Score (GSS), ranging from 0 to 24. Both SPAQ and MEQ were filled in before the study entry.

The week measured before and the week measured after the transitions were used for analysis. Sleep efficiency (actual sleep time divided by time in bed), sleep latency, actual sleep time (assumed sleep minus wake time), actual wake time, mean score in active periods, mean length of immobility, the movement and fragmentation index, bedtime and get-up time were analyzed with the software provided by the manufacturer (The Actiwatch Sleep Analysis 2001 software). Relative amplitude, intra-daily variability, and intra-daily stability were assessed using the non-parametric circadian rhythm analysis. The circadian period was assessed using fast Fourier transform analysis. All these variables were calculated for the weekdays (Monday to Thursday) before and for those after the transitions. We excluded the weekend (Friday to Sunday) from analysis. The participants kept a sleep diary. Each morning the participants marked down the time of awakening on that morning and the time of falling asleep the night before. Sleep diaries were used as assistance while doing the actigraphic analysis. Naps were scored using the Actiwatch Sleep Analysis software.

Five of the variables (the movement and fragmentation index, sleep efficiency, relative amplitude, intra-daily variability, intra-daily stability) were considered the outcome measures as decided a priori. Statistical significance was tested using non-parametric tests for two related samples [17]. Because of multiple tests, we counted a conservative Bonferroni correction (0.05 divided by 5) and considered the P values of <0.01 to be significant and those of >0.01 to <0.05 to be indicative of significance.

## Authors' contributions

TAL made contributions to the analysis and interpretation of data and to the drafting and writing of the manuscript. SL participated in the planning of the study, in the analysis of data, and in the drafting of the manuscript. JL participated in the planning of the study and in the drafting of the manuscript. TP participated in the planning of the study, in the analysis of data, and in the drafting of the manuscript. All authors read and approved the manuscript.
